# Mesenchymal Stromal Cell-Derived Extracellular Vesicles in Wound Healing

**DOI:** 10.3390/life12111733

**Published:** 2022-10-28

**Authors:** Arulkumar Nallakumarasamy, Madhan Jeyaraman, Nicola Maffulli, Naveen Jeyaraman, Veerasivabalan Suresh, Srinath Ravichandran, Manu Gupta, Anish G. Potty, Saadiq F. El-Amin, Manish Khanna, Ashim Gupta

**Affiliations:** 1 Department of Orthopaedics, All India Institute of Medical Sciences, Bhubaneswar 751019, Odissa, India; 2 Fellow in Orthopaedic Rheumatology, Dr. RML National Law University, Lucknow 226010, Uttar Pradesh, India; 3 Indian Stem Cell Study Group (ISCSG) Association, Lucknow 226010, Uttar Pradesh, India; 4 Department of Orthopaedics, Faculty of Medicine—Sri Lalithambigai Medical College and Hospital, Dr. MGR Educational and Research Institute, Chennai 600095, Tamil Nadu, India; 5 Department of Medical Research and Translational Medicine, Faculty of Medicine—Sri Lalithambigai Medical College and Hospital, Dr. MGR Educational and Research Institute, Chennai 600095, Tamil Nadu, India; 6 Department of Biotechnology, School of Engineering and Technology, Sharda University, Greater Noida 201310, Uttar Pradesh, India; 7 South Texas Orthopaedic Research Institute (STORI Inc.), Laredo, TX 78045, USA; 8 Department of Musculoskeletal Disorders, School of Medicine and Surgery, University of Salerno, 84084 Fisciano, Italy; 9 San Giovanni di Dio e Ruggi D’Aragona Hospital “Clinica Ortopedica” Department, Hospital of Salerno, 84124 Salerno, Italy; 10 Barts and the London School of Medicine and Dentistry, Centre for Sports and Exercise Medicine, Queen Mary University of London, London E1 4DG, UK; 11 School of Pharmacy and Bioengineering, Keele University School of Medicine, Stoke on Trent ST5 5BG, UK; 12 Fellow in Joint Replacement, Department of Orthopaedics, Atlas Hospitals, Tiruchirappalli 620002, Tamil Nadu, India; 13 Department of Obstetrics-Gynecology, Madras Medical College and Hospital, Chennai 600003, Tamil Nadu, India; 14 Department of General and GI Surgery, Stepping Hill Hospital, Stockport NHS Foundation Trust, Stockport SK27JE, UK; 15 Polar Aesthetics Dental & Cosmetic Centre, Noida 201301, Uttar Pradesh, India; 16 El-Amin Orthopaedic & Sports Medicine Institute, Lawrenceville, GA 30043, USA; 17 Regenerative Sports Medicine, Lawrenceville, GA 30043, USA; 18 BioIntegrate, Lawrenceville, GA 30043, USA; 19 Department of Orthopaedics, Autonomous State Medical College, Ayodhya 224135, Uttar Pradesh, India; 20 Regenerative Orthopaedics, Noida 201301, Uttar Pradesh, India; 21 Future Biologics, Lawrenceville, GA 30043, USA

**Keywords:** extracellular vesicles, exosomes, chronic wounds, diabetic wounds, mesenchymal stromal cells, mesenchymal stem cells, hydrogels, regenerative medicine

## Abstract

The well-orchestrated process of wound healing may be negatively impacted from interrupted or incomplete tissue regenerative processes. The healing potential is further compromised in patients with diabetes mellitus, chronic venous insufficiency, critical limb ischemia, and immunocompromised conditions, with a high health care burden and expenditure. Stem cell-based therapy has shown promising results in clinical studies. Mesenchymal stem cell-derived exosomes (MSC Exos) may favorably impact intercellular signaling and immunomodulation, promoting neoangiogenesis, collagen synthesis, and neoepithelization. This article gives an outline of the biogenesis and mechanism of extracellular vesicles (EVs), particularly exosomes, in the process of tissue regeneration and discusses the use of preconditioned exosomes, platelet-rich plasma-derived exosomes, and engineered exosomes in three-dimensional bioscaffolds such as hydrogels (collagen and chitosan) to prolong the contact time of exosomes at the recipient site within the target tissue. An appropriate antibiotic therapy based on culture-specific guidance coupled with the knowledge of biopolymers helps to fabricate nanotherapeutic materials loaded with MSC Exos to effectively deliver drugs locally and promote novel approaches for the management of chronic wounds.

## 1. Introduction

Extracellular vesicles (EVs) are cell-specific lipid-bound organelles that facilitate intercellular communication with their cargo elements, including proteins, nucleic acids, and certain lipids [1]. Various types of EVs have been described, including ectosomes, microvesicles, microparticles, exosomes, oncosomes, apoptotic bodies, and exomeres [2,3]. Exosomes are a nanosized clinically relevant EV type with diagnostic and therapeutic applications [4,5,6]. The regulated biogenesis of exosomes and the specific targeting action of cell-specific cargo materials over the recipient cells are of interest in the field of immunological disorders and regenerative medicine [7,8,9].

**Figure 1:**
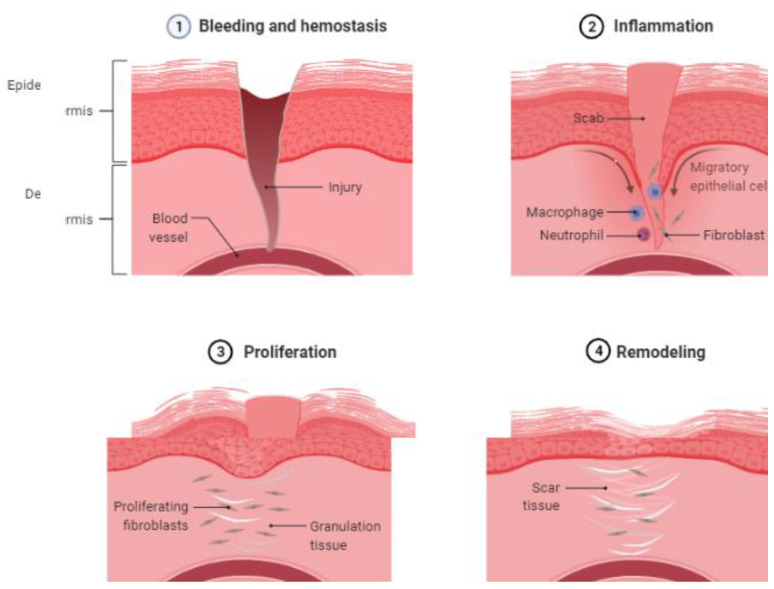
Natural course of wound healing. Four phases of wound healing (1) bleeding and hemostasis, (2) inflammation, (3) proliferation, and (4) remodeling. (created with BioRender.com).

The process of wound healing has four phases: (a) hemostasis; (b) inflammatory; (c) proliferative; and (d) remodeling (Figure 1) [10,11,12,13,14,15].

*(a)*  *Hemostasis*: The earliest phase in wound healing starts with the formation of a platelet plug and the activation of the coagulation cascade to reduce bleeding. Platelets are activated when they come into contact with extracellular collagen, releasing growth factors that cause platelet aggregation and clumping. This is followed by the activation of the coagulation cascade.
*(b)*  *Inflammation*: This phase begins within 24 h of the injury and lasts up to 2 weeks, first with the recruitment of neutrophils and then macrophages. These cells release various cytokines (IL-1, -6, -8, and TNF-alpha) and growth factors (PDGF, TGF-beta, TGF-alpha, and fibroblast growth factors) to activate fibroblasts and epithelial cells. Neutrophils serve as the first line of defense. Macrophages are activated later. The classical proinflammatory pathway of macrophages is activated first, followed by the alternate macrophage pathway (M2).
*(c)*  *Proliferative* phase: This phase is characterized by fibroblast migration, collagen and extracellular matrix production, angiogenesis, the laying of granulation tissue, and epithelialization. Fibroblasts begin moving by first binding to the matrix components (such as fibronectin) via the integrity receptors. The direction of the fibroblast movement is determined by the concentration gradient of cytokines and growth factors. Fibroblasts secrete matrix metalloproteinase, collagenase, and gelatinase to degrade the extracellular matrix, facilitating cell migration and movement. After fibroblast migration, there is an increased production of the extracellular matrix through stimulation by TGF-β and PDGF. Damaged vasculature must be replaced by new blood vessels through angiogenesis, stimulated by VEGF, HIF, and PEGF. In epithelialization, epithelial cells around the margin of the wound lose contact inhibition and epiboly migrate into the wound area [16].
*(d)*  *Remodeling*: This is the final phase of the healing process, with the formation of granulation tissue. Type 3 collagen is gradually replaced by type 1 collagen.

This review provides a brief outline of the natural processes of wound healing as well as the morphology, biogenesis, and applications of exosomes. In addition, the mechanism and molecular signaling targets of exosomes in the management of chronic non-healing wounds are described. Finally, we discuss the production of engineered bioscaffolds with functionalized nanovesicles to use in chronic wounds.

## 2. Forms and Functions of Extracellular Vesicles

**Figure 2:**
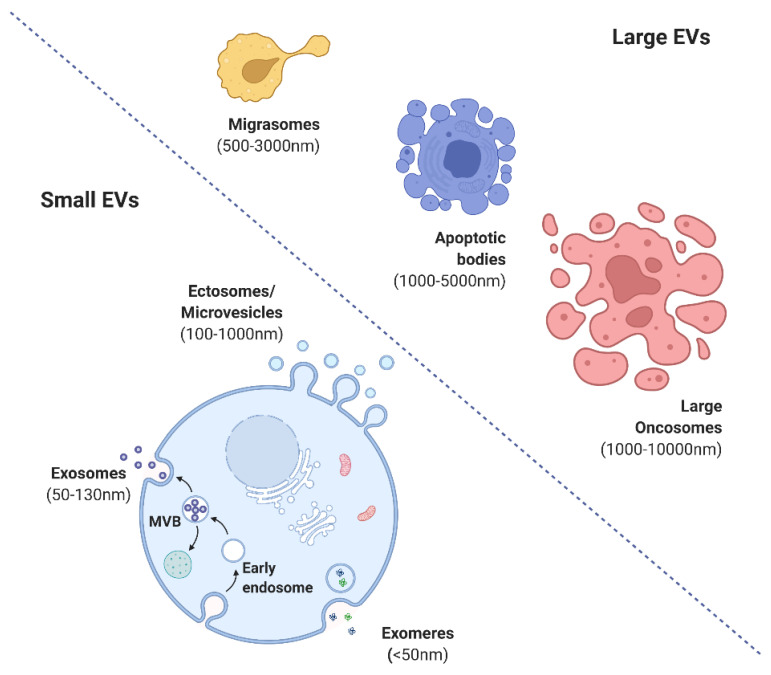
Various forms of extracellular vesicles (created with BioRender.com).

The International Society for Extracellular Vesicles (ISEV) has proposed guidelines for the nomenclature, isolation, and characterization of EVs. EVs are broadly categorized into small (exomeres (<50 mm), exosomes (<100 or 200 nm), and ectosomes (>200 nm) or shedding microvesicles (MVs)) and large (migrasomes (500–3000 nm), apoptotic bodies (1000–5000 nm), and large oncosomes (1000–10,000 nm)) (Figure 2) [4,17]. Exosomes evolve by sprouting as intraluminal vesicles (ILVs) within the luminal space of late endosomes or so-called multivesicular bodies (MVBs). They are produced and released by various cells, tissues, and body fluids [8]. EVs are involved in cell-to-cell interaction pathways with physiological and pathological functions [18,19]. EVs possess immunomodulatory and immunosuppressive effects and activate angiogenesis, the proliferative phase, and epithelialization.

## 3. Biogenesis of MSC-Derived EVs

In this field, the best-known mechanism is probably the endosomal sorting complex required for transport (ESCRT) [20,21]. The cascade promotes the activation of rho-associated protein kinase 1 (ROCK-1). ROCK-1 phosphorylates the myosin regulatory light chain and stimulates the contractile activity of actomyosin. It then leads to the formation of apoptotic bodies. Exosomes are formed during endosomal sorting. Intraluminal vesicles mature into ESCRT [22,23]. Microvesicle biogenesis involves the calcium-dependent enzymes calpain, gelsolin, phospholipid translocase, and scramblase [6,24].

The preconditioning of MSCs is performed in the first 24 h after harvesting. This period of incubation alters the cell microenvironment by inducing hypoxia, oxidative stress, and inflammation. Four cycles of ultracentrifugation are required to isolate the extracellular vesicles from the preconditioned MSCs. At the end of the first spin, the cell debris is formed; after the second spin, large-sized extracellular vesicles are collected. After the third spin, medium-sized extracellular vesicles are collected and in the last spin, small-sized extracellular vesicles are obtained [25,26,27]. Various techniques are available to assess the regulation of EVs, namely: (a) a scratch wound assay (used to study cell growth and healing and especially useful to study wound closure rates and tracking wound closures for 24 h) [28]; (b) a nanoparticle tracking analysis (performed in real time to quantify exosomes in the range of 50 to 1000 nm in a liquid suspension) [29]; (c) dynamic light scattering (using scattered light from the Brownian motion of particles to determine the particle concentration and size) [30]; (d) electron microscopy (scanning electron microscopy (SEM) and transmission electron microscopy (TEM) can be used to analyze the morphology of exosomes) [31]; (e) tunable resistive pulse sensing (tRPS) (fluid is divided into two halves by a non-conductive nanomembrane. One half contains a suspension and the other half contains a particle-free electrolyte. An electric potential is applied and a resistive pulse is generated. The length of the pulse is proportional to the particle size) [32]; and (f) cell number recovery (CNR) (the ratio of cells in the wound region at time t to cells in the wound region at time 0) [33,34]. Other biochemical methods such as Western blotting, size-exclusion chromatography, flow cytometry, and thermophoretic profiling can also be used to analyze extracellular vesicles [33,35,36,37,38,39].

## 4. Molecular Signaling Targets of EVs in Wound Healing

### 4.1. EVs in Hemostasis through Glycoproteins and Oxidases

Platelet-derived extracellular vesicles are most abundant in the circulation and help to activate platelets and the formation of fibrin clots. Platelet-derived extracellular vesicles activate both extrinsic and intrinsic pathways [40]. They indirectly exert procoagulant effects by binding P-selectin to P-selectin glycoprotein ligand-1 (PSGL1) [40]. Platelet-derived extracellular vesicles can also interact with NADPH oxidase (NOX) [41]. They are involved in superoxide generation and enhance fibrin binding. Platelet-derived extracellular vesicles also induce platelet activation by collagen receptors [42,43]. UC-MSC-derived EVs suppress ROS-induced apoptosis through the suppression of AIF nuclear translocation and PARP-1 activation [44].

### 4.2. EVs in Inflammation through Adhesion Molecules and ROS Products

Neutrophil-derived extracellular vesicles (NDEVs) show anti-inflammatory and proinflammatory functions, depending on environmental factors [45,46]. They increase the expression of adhesion molecules such as E-selectin and VCAM 1 and increase ROS production. NDEVs mediate inflammation by producing danger signals. Endothelium-attached NDEVs induce proinflammatory genes whereas non-adherent NDEVs induce anti-inflammatory genes [45]. During the inflammatory phase, macrophages play an important role in the transition from the inflammatory phase to the proliferative phase. Macrophage-derived EVs induce the reprogramming of macrophages from the M1 to the M2 phenotype [47,48]. Extracellular vesicles derived from keratinocytes from the wound edge also cause a similar phenotype change in macrophages [49]. M2 extracellular vesicles decrease the expression of M1 marker iNOS but increase the expression of arginase, an M2 macrophage marker [49].

The TLR4/NF-κB/STAT3/AKT regulatory signaling pathway plays a critical role in the regulation of macrophage plasticity [50]. LPS-preconditioned UC-MSCs modify macrophage polarization for the resolution of chronic inflammation via Exos-shuttled let-7b [51]. Macrophage reactivity, polarization, and modulation in the wound led by MSC-derived EVs are facilitated by the transfer of miRNAs such as let-7b and -181c, which results in the downregulation of proinflammatory (TNF-α and IL-1β) micromolecules and the upregulation of anti-inflammatory (TGF-β and IL-10) micromolecules [51,52]. Glycolysis is the source of energy for proinflammatory M1 macrophages by inhibiting mitochondrial oxidative phosphorylation and the TCA cycle whereas mitochondrial oxidative phosphorylation is the energy feeder for anti-inflammatory M2 macrophages [53].

Human BM-MSC EVs, when administered as an IV injection at the wound site, promote wound healing and the polarization of macrophages to the M2 phenotype. EVs in vitro human monocytes/macrophages promote M2 macrophage polarization through the transfer of miR-223 [54]. Melatonin-stimulated BM-MSC-derived EVs improve diabetic wound healing through regulating macrophage polarization by targeting the PTEN/AKT pathway [55].

### 4.3. EVs in Proliferation and the Mechanism in Wound Healing

EVs derived from umbilical progenitor cells have proangiogenic effects [56]. They stimulate angiogenesis through the modulation of the AKT/ERK/STAT 3 pathway, modulation of the NOTCH pathway, increased expression of miR-126, and stimulation of the WNT/beta-catenin pathway [56]. Treg cells play a significant role in the healing of the wound bed. Tissue-resident Treg cells provide a conductive environment for proper wound healing through the amphiregulin-TGF-β cascade [57,58,59,60]. γδTreg cells secrete KGF and IGF-1 to promote the proliferation and survival of keratinocyte [61]. The upregulation of OCT-4 and NANOG expression and the downregulation of vinculin were observed when MSCs were incubated along with MSC-derived EVs. Such a combination delays premature senescence, facilitates stemness, and enhances glycolytic metabolism in MSCs via the activation of miR-302b and HIF-1α [62].

BM-MSC-derived Exos accelerate wound healing by targeting fibroblasts via the Akt, Erk1/2, and STAT3 signaling pathways [63]. FGF-2, IL-6, and -8 upregulate the Erk1/2 pathway, which results in cellular proliferation, migration, and angiogenesis [64,65,66] whereas Id-1, Cox-2, VEGFA, and c-myc upregulate the Erk1/2 pathway at the mRNA level [67,68,69,70]. Mouse BM-MSC-derived EVs promoted the proliferation, migration, and tube formation of in vitro endothelial cells and increased the p-AKT and p-eNOS signaling pathways to produce angiogenesis in a healing wound [71]. BM-MSC-derived Exos lncRNA H19 promoted wound healing in diabetic foot ulcers by upregulating PTEN via miRNA-152-3p [72]. BM-MSC-derived EVs are rich in proliferative factors (the proliferation and promotion of the viability of keratinocytes, fibroblasts, and endothelial cells) whereas AD-MSC-derived EVs are rich in proangiogenic factors (the proliferation of endothelial cells) [73]. Enhanced vasculogenesis was observed in wound beds when hBM-MSC-derived EVs were stimulated by deferoxamine. The combination of deferoxamine and Exos activated the PI3K/AKT signaling pathway via miR-126-mediated PTEN downregulation to stimulate angiogenesis in vitro [74]. Exos derived from atorvastatin-pretreated BM-MSCs accelerated diabetic wound repair by enhancing angiogenesis via the AKT/eNOS pathway by upregulating miR-221-3p in endothelial cells [75]. A static magnetic field-induced BM-MSC-derived Exos promoted neovasculogenesis to enhance wound healing through miR-21-5p by targeting SPRY2 to facilitate the PI3K/AKT and ERK1/2 signaling pathways [76].

Ren et al. demonstrated that AD-MSC-derived EVs facilitate wound healing via the AKT serine/threonine kinase 1 (AKT) and mitogen-activated protein kinase 1 (ERK) signaling pathways [77]. These AD-MSC-derived EVs upregulate cyclin D1, D2, A1, and A2 as well as growth factors (VEGF-A, PDGF-A, EGF, and FGF-2); hence, they increase wound epithelialization, collagen synthesis, angiogenesis, and wound contracture [77]. AD-MSC-derived Exos accelerate wound healing through the PI3K/AKT signaling pathway [78]. AD-MSC-derived EVs promote the proliferation and migration—and stimulate the AKT and ERK signaling—of in vitro fibroblasts, keratinocytes, and endothelial cells [77]. AD-MSC-derived EVs facilitate wound healing by accelerating keratinocytes and fibroblasts in an AKT/HIF-1α-dependent fashion [79]. Human AD-MSC-derived EVs attenuate hypertrophic scars and fibrosis by the miR-192-5p/IL-17RA/SMAD axis [80]. Exos derived from mmu_circ_0000250-modified AD-MSCs promoted wound healing in diabetic mice by inducing miR-128-3p/SIRT1-mediated autophagy [14]. The overexpression of Nrf2 by AD-MSC-derived EVs facilitated wound healing by enhancing neovasculogenesis in a diabetic foot ulcer in a rat model. Nrf2 overexpression leads to the robust formation of granulation tissue, neovasculogenesis, and the raised expression of growth factors as well as decreased levels of proinflammatory molecules and oxidative stress-related proteins in wound beds [81].

In dermal fibroblasts and in a keratinocyte-deficient wound model, UC-MSC-derived EVs promoted the proliferation and migration of fibroblasts and keratinocytes through miR-27b, which acts by suppressing E3-ubiquitin ligase ITCH, thereby activating JUNB/IRE1α [82]. UC-MSC-derived Exos enhance wound healing by activating the WNT/β-catenin signaling pathway [83]. Endothelial progenitor cell (EPC)-derived Exos accelerate wound healing by facilitating vasculogenesis via Erk1/2 signaling [84]. UC-MSC-derived EVs, along with a pluronic F127 hydrogel, facilitated wound healing by promoting neovasculogenesis and the increased expression of VEGF and TGF-β1 [85].

Apoptotic body (AB)-derived EVs accelerate cutaneous wound healing, promote macrophage M2 polarization through downregulated iNOS activity and an upregulated ARG:iNOS ratio, and enhance the functions of fibroblasts and keratinocytes in the wound healing pathway. Given the lack of standardization, cell sources, and retention rate of MSCs after transplantation, AB-derived EVs provide a promising platform to develop a cell-free therapy [86].

### 4.4. EVs in the Remodeling of Wound Healing

EVs facilitate collagen 1 cross-linking and promote collagen gel contraction. A few components of fibrocyte-derived EVs (FDEVs) such as hsp-90 alpha and STAT-3 promote cell motility and re-epithelialization [45,87,88]. FDEVs are also rich in anti-inflammatory miRNAs such as miR124a and miR125b [89]. MSC-derived EVs enhance the re-epithelialization, neovasculogenesis, proliferation, and migration of cellular components to the injured site by increasing MMP-9, PDGF-A, VEGF-A, FGF-2, TGF-β, and EGF and modulating the NOTCH, AKT/ERK, and WNT/β-catenin signaling pathways, enhancing the production of collagen 1 and 3, fibronectin, and extracellular matrix components [90,91,92,93,94,95]. Human fetal dermis-bound MSC-derived Exos induce the expression of COL1, COL3, elastin, and fibronectin by activating the NOTCH pathway [96].

TSG-6-modified BM-MSC-derived EVs suppress scar formation by suppressing SMAD2/3 signaling and by inhibiting TGF-β1, COL1, COL3, and SMA-α protein synthesis and inflammation in the wound site [97]. A local injection of EVs improves wound healing by increasing the mRNA for COL1 and COL3 as well as the mRNA for N-cadherin and elastin [98]. An IV injection of AD-MSC-derived EVs migrates to the wound site and spleen, promoting wound healing [98]. In vitro fibroblasts in response to AD-MSC-derived EVs promote the proliferation and migration of fibroblasts and keratinocytes and receive signals from COL1, COL3, MMP1, FGF2, and TGF-β1 mRNAs along with the increased expression of VEGF, c-myc, MMP-9, and fibronectin [77,99]. The application of PI3K/AKT inhibitor Ly294002 abrogated the EV-induced effects of fibroblasts on a wound surface [78].

AD-MSC-derived Exos facilitate extracellular matrix remodulation in wound repairs by enhancing and regulating the COL3:1, TGF-β3:TGF-β1, and MMP3:TIMP1 ratios via the ERK/MAPK signaling pathways to mitigate the minimization of scar formation [100]. The promotion of wound healing by AD-MSC-derived Exos/EVs is facilitated by the overexpression of miR-486-5P by targeting Sp5 and CCND2 expression [101]. The overexpression of miR-135a downregulates LATS2; hence, it upregulates cellular migration and enhances wound healing [102]. AD-MSC-derived miR-192-5p downregulates pro-fibrosis protein and upregulates wound healing via the inhibition of IL-17RA/SMAD expression [80].

**Figure 3:**
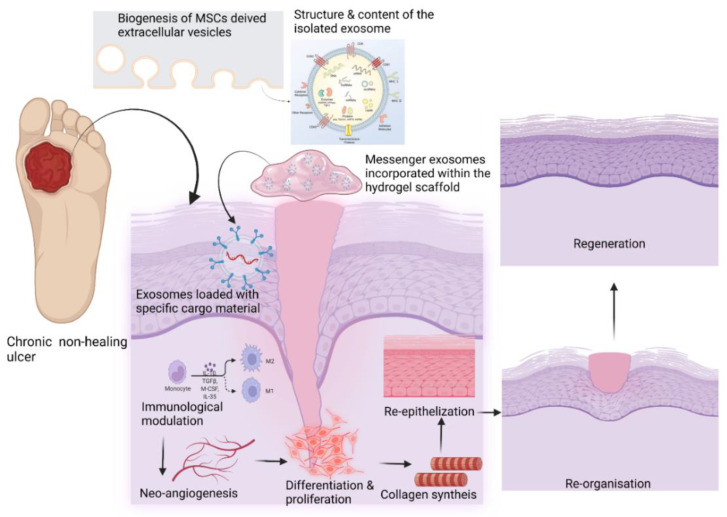
Role of EVs in wound healing (created with BioRender.com).

UC-MSC-derived EVs suppressed TGF-β-induced myofibroblast formation in a mouse skin wound model. These EVs were enriched with miR-21, -23a, -125b, and -145, which reduced the TGF-β/SMAD2 signaling in the fibroblasts [103]. An accelerated re-epithelization of burned skin on rats was observed with the administration of UC-MSC-derived Exos via Wnt-4 signaling [83]. In a skin defect mouse model, UC-MSC-derived Exos inhibited myofibroblast differentiation by suppressing the TGF-β2/SMAD2 pathway through miRNAs (miR-21, -23a, -125b, and -145), which resulted in reduced fibrosis and scar formation [103,104]. Amniotic fluid-MSC-derived EVs inhibited and suppressed myofibroblast aggregation and ECM synthesis via the TGF-β pathway through miRNAs such as let-7-5p, -22-3p, -27a-3p, -21-5p, and -23a-3p [105]. UC-MSC-derived Exos promoted the phosphorylation of YAP by transporting the 14-3-3*ζ* protein, which inhibited WNT/β-catenin signal transduction, enhanced collagen deposition, and inhibited excess fibroblast expansion in burn wounds. Such mechanisms have improved tissue remodeling and reduced scar formation in burn wounds [106]. MSC-derived EVs act by targeting the injured site by producing scarless re-epithelialization and decreasing cell senescence (Figure 3) [107,108]. A summary of the role of MSC-derived EVs is described in Table 1.

## 5. New Perspectives of EV-based Therapy in Wound Healing

EVs have been classically thought to be vestigial and have been poorly investigated for regenerative medicine purposes. Pluripotent nanovesicles can be retrieved from MSCs of various origins (bone marrow, placenta, umbilical cord, and adipose tissue-derived) and endothelial progenitor cells, and only recently have MSC-derived EVs been more intensively studied for their application in tissue-specific regenerative medicine. Intercellular communication and the specific tissue targeting effect mediated by these nanobiomolecules play a role in immunomodulation, angiogenesis, the amplification of the growth potential, and regeneration [109,110,111,112,113]. The proteomic and transcriptomic analysis of EVs with loaded bioactive cargo molecules provides an interesting bioprofile regarding their diagnostic and therapeutic efficacy.

### 5.1. Engineered EV Therapy

The wound healing tendency in immunocompromised conditions such as diabetes mellitus and chronic kidney disease is negatively impacted by an impaired local immunity, which leads to a prolonged inflammatory phase and poor vascularity. In such conditions, the priming and recruitment of neutrophils are compromised and innate immunity tends to be defective. In these chronic wounds, engineered EVs have the potential to improve the chemotactic response, activate the respiratory burst of the neutrophils, and facilitate neoangiogenesis and site-specific tissue differentiation, promoting healing [114]. EV therapy yields many advantages over cell-based therapies, including immunocompatibility, no shear stress following an injectable therapy, and a non-carcinogenic growth potential. Loco-regional angiogenesis accelerates collagen synthesis and full-thickness wound healing and improves the quality of the scar formation [115,116]. MSC EVs can be administered via an intravenous route, a direct injection, or a topical application. Injected MSCs exert their physiological effect at the recipient site by their paracrine secretion of extracellular vesicles rather than a direct differentiation [117].

### 5.2. EV-Induced Immunomodulation

Macrophages are involved in phagocytosis and the process of tissue healing. They are phenotypically classified into M1 (classically activated) and M2 (alternatively activated) macrophages. Studies have shown improved wound healing after the administration of bone marrow-derived MSCs to a wound site by promoting M2 polarization [54]. Polarized M2 macrophages induce the secretion of chemokines such as TNF-α, IFN-γ, and IL-1 and mediate the surge of VEGF, PDGF, and TGF-β into the local environment. Thus, the correct temporal sequence of the M1 to M2 shift mediated by EVs is important in the treatment of chronic wounds.

### 5.3. PRP-Derived EV Therapy

The activation of the Hippo/YAP (Yes-associated protein) signal pathway is essential for the process of epidermal re-epithelization. Platelet-rich plasma contains various growth factors essential for wound healing. EVs derived from PRP show benefits for tissue regeneration by activating the YAP pathway [118]. A PRP-derived exosomal therapy can accelerate the process of collagen deposition in wound beds. When analyzing the dose-dependent therapeutic efficacy of platelet lysate-derived exosomes, the isolated exosomes were shown to contain a higher amount of essential growth factors (βFGF, VEGF, PDGF-BB, and TGF-β1) and small RNAs compared with the donor platelets [119].

### 5.4. Bioscaffolds with Functionalized EV Therapy

Most commonly, EVs are delivered via a direct injection at the desired site. However, this can impair the function because of rapid metabolic clearance. Although MSC-derived exosomes have great potential in disease treatment, issues such as rapid clearance and the maintenance of their inadequate preservation for their viability and function remain to be addressed [120,121]. To date, there is no effective method to retain retrieved MSC-based EVs at the wound site. Thus, tissue-engineered biocompatible scaffold constructs provide the skeletal framework for the extracellular vesicles at the desired site to exert their prolonged therapeutic effect of healing and regeneration [122,123]. However, many studies have recently reported that these traditional scaffolds lack the porous structure needed for cell growth, proliferation, and migration [124,125]. Liu et al. designed a hydrogel glue that could retain stem cell-derived exosomes (SC Exos) to enhance the chondrogenic potential at the defect area [126]. Furthermore, they suggested that this novel acellular exosome-rich hydrogel glue (EHG) could be used as scaffold material for tissue regeneration in chronic wounds.

Chronic diabetic wounds require a relatively long time for tissue regeneration and healing. Wang et al. developed a novel stem cell-derived exosome-rich biocompatible scaffold that could serve as a sustained release of growth factors with a local immunomodulation to maintain their bioactivity at the wound site. This is more advantageous than the previously used injection technique, given its enhanced bioavailability and its anchorage to the surrounding tissue [127]. Recent research has investigated the superior angiogenic properties in wound repair using bone marrow-derived MSCs (BMSCs) preconditioned by dimethyloxaloyl glycine [128], pioglitazone [128], deferoxamine (DFO Exos) [74], and atorvastatin [75] in animal models.

Hydrogels of thiolated chitosan (CSS) and polyethylene glycol-maleimide-modified ε-polylysine (EPL-PEG-MAL) showed enhanced elastic and adhesive properties and no cytotoxicity. These scaffolds with EVs have been developed to accelerate angiogenesis, tissue regeneration, and wound healing [129]. However, the development of an ideal hydrogel scaffold composed of natural polypeptides with biocompatibility and antibacterial properties for EV delivery and tissue regeneration is still in the early stages [130].

Li et al. constructed a genipin cross-linked dual-sensitive hydrogel loaded with human umbilical cord-derived MSC (hUC-MSC)-derived EVs to boost cutaneous wound healing. The authors showed accelerated collagen deposition, increased epidermal re-epithelialization rates, and early wound closure [131]. In addition, Yang et al. reported that they constructed F127 hydrogels as release carriers for exosomes; the exosomes released from the hydrogel markedly improved skin regeneration and promoted wound healing compared with the exosomes-alone group [85]. Salivary peptides such as histatin-1 accelerate wound healing, but their mechanism of action is still unknown. In vitro and in vivo studies have shown that saliva Exos were the source of an ubiquitin-conjugating enzyme E2O (UBE2O) type of mRNA. The activation of UBE2O decreased the expression of SMAD6 and increased the expression of BMP2, which, in turn, induced angiogenesis and accelerated wound healing [132].

Recently, researchers constructed a 3D scaffold composed of decellularized small intestinal submucosa (SIS) combined with a mesoporous bioactive glass (MBG), which facilitated the sustained release of BMSC-derived exosomes. They induced diabetes in rats by injecting streptozocin and then produced a 1.5 cm full-thickness skin wound. A cryogenic 3D SIS/MBG@Exos hydrogel scaffold was structured according to the size of the wound and they covered the wound with that scaffolding material appropriately. The result showed neoangiogenesis with patterned collagen deposition, the early formation of granulation tissue, and accelerated full-thickness wound healing in the control group [133]. Split-skin grafts and flap surgeries are widely used to cover the surface of chronic wounds once they are germ-free, but graft failure is not uncommon; an early failure may lead to wound dehiscence and further complications. In vivo, adipose mesenchymal stem cell exosomes preconditioned with H_2_O_2_ (H_2_O_2_-ADSC Exos) reduced apoptosis and increased the capillary density and blood perfusion unit (BPU) of the graft. Therefore, they significantly increased the flap survival rate following an ischemia/reperfusion (I/R) injury [134].

Currently, none of the extracellular vesicle products have been approved by the FDA for therapeutic use in humans. The FDA has only approved umbilical cord-derived stem cells for treating certain hematopoietic disorders and issued a Public Safety Notification in June 2019 to prevent the manufacturing or marketing of illegal “stem cell” products [135]. Further guidelines to regulate regenerative medicine products were issued in September 2021 [136].

Most of the studies to date were preclinical. The results of two clinical trials evaluating the efficacy of serum-derived EVs in wound therapy are still awaited [137,138]. Therefore, future generations need to gain a deeper knowledge about the biogenesis, isolation, action mechanisms, therapeutic dose, mode of administration, and complications of EV therapy to effectively treat chronic wounds.

## 6. Conclusions

Full-thickness cutaneous wound therapy and regeneration remain major clinical challenges. Wound healing is a highly integrated complex biological process that involves hemostasis, immunomodulation, intercellular communication, and the well-orchestrated release of various growth factors. This combined effect of local molecular homeostasis eventually induces collagen deposition and tissue regeneration. Stem cell-derived EVs can build up a pro-healing environment by activating intercellular signaling, angiogenesis, proliferation, and the regional differentiation of the various cell types in tissue regeneration.

In addition, the development of 3D printing technologies can help to fabricate size-specific functional scaffolds to be used in the treatment of chronic non-healing wounds. Continued advances in controlled drug delivery using MSC EVs should allow for the development of new highly effective loco-regional antibiotic delivery strategies. However, the therapeutic benefits of MSC EVs need to be proven in large-scale clinical studies to test the efficacy of such novel treatment modalities.

## Figures and Tables

**Table 1:** Summary of MSC-derived EVs in wound healing.

Form of MSC	Extracellular Vesicles	Significance
	BM-MSC EVs	Promote M2 macrophage polarization through transfer of miR-223. Macrophage polarization by targeting the PTEN/AKT pathway. Promote proliferation, migration, and tube formation of in vitro endothelial cells and increase the p-AKT and p-eNOS signaling pathways to produce angiogenesis in the healing wound.
	BM-MSC Exos	Accelerate wound healing by targeting fibroblasts via the Akt, Erk1/2, and STAT3 signaling pathways.
	BM-MSC Exos lncRNA H19	Promotes wound healing in diabetic foot ulcers by upregulating PTEN via miRNA-152-3p.
	BM-MSC Exos + deferoxamine	Activate the PI3K/AKT signaling pathway via miR-126-mediated PTEN downregulation to stimulate angiogenesis in vitro.
	BM-MSC Exos + atorvastatin	Enhance angiogenesis via the AKT/eNOS pathway by upregulating miR-221-3p in endothelial cells.
	BM-MSC Exos + static magnetic field	Promote neovasculogenesis to enhance wound healing through miR-21-5p by targeting SPRY2 to facilitate the PI3K/AKT and ERK1/2 signaling pathways.
	TSG-6-modified BM-MSC EVs	Suppress scar formation by suppressing SMAD2/3 signaling and inhibiting TGF-β1, COL1, COL3, and SMA-α protein synthesis and inflammation in the wound site.
Adipose tissue-derived MSCs	AD-MSC EVs	Facilitate wound healing via the AKT serine/threonine kinase 1 (AKT) and mitogen-activated protein kinase 1 (ERK) signaling pathways. Promote the proliferation and migration—and stimulate the AKT and ERK signaling—of in vitro fibroblasts, keratinocytes, and endothelial cells. Facilitate wound healing by accelerating keratinocytes and fibroblasts in an AKT/HIF-1α-dependent fashion. Attenuate hypertrophic scars and fibrosis by the miR-192-5p/IL-17RA/SMAD axis. Promote the proliferation and migration of fibroblasts and keratinocytes, receive signals from COL1, COL3, MMP1, FGF-2, and TGF-β1 mRNAs, along with the increased expression of VEGF, c-myc, MMP-9, and fibronectin.
	AD-MSC Exos	Accelerate wound healing through the PI3K/AKT signaling pathway. Facilitate extracellular matrix remodulation in wound repair by enhancing and regulating the COL3:1, TGF-β3:TGF-β1, and MMP3:TIMP1 ratios via the ERK/MAPK signaling pathways to mitigate the minimization of scar formation. Facilitate wound healing by the overexpression of miR-486-5p by targeting the Sp5 and CCND2 expression.
	Exos derived from mmu_circ_0000250-modified AD-MSCs	Promoted wound healing in diabetic mice by inducing miR-128-3p/SIRT1-mediated autophagy.
	AD-MSC-derived miR-192-5p	Downregulates pro-fibrosis protein and upregulates wound healing via the inhibition of the IL-17RA/SMAD expression.
Umbilical cord-derived MSCs	UC-MSC EVs	Promote the proliferation and migration of fibroblasts and keratinocytes through miR-27b, which acts by suppressing E3-ubiquitin ligase ITCH, thereby activating JUNB/IRE1α. Suppressed TGF-β-induced myofibroblast formation in a mouse skin wound model through TGF-β/SMAD2 signaling in fibroblasts.
	UC-MSC Exos	Enhance wound healing by activating the WNT/β-catenin signaling pathway. Promote the phosphorylation of YAP by transporting the 14-3-3*ζ* protein, which inhibited WNT/β-catenin signal transduction, enhanced collagen deposition, and inhibited excess fibroblast expansion in burn wounds.
	UC-MSC Exos + pluronic F127 hydrogel	Promote neovasculogenesis by increasing the expression of VEGF and TGF-β1.
Amniotic fluid-derived MSCs	AF-MSC EVs	Inhibit and suppress myofibroblast aggregation and ECM synthesis via the TGF-β pathway through miRNAs such as let-7-5p, -22-3p, -27a-3p, -21-5p, and -23a-3p.
Human fetal dermis-derived MSCs	Human fetal dermis-MSC EVs	induce the expression of COL1, COL3, elastin, and fibronectin by activating the NOTCH pathway.

## Data Availability

The data are contained within the article.

## Abbreviations

AD-MSCs: adipose tissue-derived mesenchymal stem cells; AF-MSCs: amniotic fluid-derived mesenchymal stem cells; BM-MSCs: bone marrow-derived mesenchymal stem cells; ESCRT: endosomal sorting complex required for transport; EVs: extracellular vesicles; Exos: exosomes; ILVs: intraluminal vesicles; ISEV: International Society for Extracellular Vesicles; LPS: lipopolysaccharide; MSCs Exos: mesenchymal stem cell-derived exosomes; MVBs: multivesicular bodies; NDEVs: neutrophil-derived extracellular vesicles; ROS: reactive oxygen species; UC-MSCs: umbilical cord-derived mesenchymal stem cells.
